# Directed Evolution of Xylanase from *Dickeya dadantii* DCE-01 with Improved Enzymatic Activity

**DOI:** 10.3390/polym17192650

**Published:** 2025-09-30

**Authors:** Ruijun Wang, Ke Shi, Ke Zheng, Qi Yang, Guoguo Xi, Shengwen Duan, Lifeng Cheng

**Affiliations:** 1 School of Pharmacy, Yichun University, Yichun 336000, China; wruijun@163.com; 2 Institute of Bast Fiber Crops, Chinese Academy of Agricultural Sciences, Changsha 410205, China

**Keywords:** *Dickeya dadantii* DCE-01, xylanase, enzymatic activity, error-prone PCR

## Abstract

Xylanase, an essential enzyme for breaking down xylan, faces limitations in its industrial applications due to the relatively low catalytic activity of the wild type. Directed evolution was used to enhance the catalytic efficiency of xylanase that originated from the *Dickeya dadantii* DCE-01. A xylanase variant (Xyn-ep) was obtained with improved catalytic activity by random mutant library employing two rounds of error-prone PCR. The results showed that the Xyn-ep demonstrated enzyme activity 1.6 times higher than that of wild-type xylanase. Sequencing analysis pinpointed key mutation sites at S159P, K212N, and N397S, respectively. Homology modeling was used to analyze the location of the mutation sites and to investigate the mechanism of the improved catalytic performance. The mutant Xyn-ep showed improved catalytic performance by error-prone PCR. Additionally, the increased flexibility of the loop of the mutant may contribute to the enhanced activity. These findings indicate that error-prone PCR is an effective method for enhancing enzyme activity and that the mutant Xyn-ep may be a new GH30 xylanase, being a potential candidate for industrial applications such as bast fiber bio-degumming, cotton bio-refinery, paper making, and so on.

## 1. Introduction

Xylan, a major polysaccharide in plant cell walls, is a renewable resource second only to cellulose in natural abundance [1]. It is a high-molecular-weight polymer with a complex chemical composition. The xylan backbone is mainly composed of xylose residues connected by β-D-1,4-glycosidic bonds [2]. However, xylans from different plants may contain distinct side chains, such as acetyl, arabinosyl, galactosyl, glucuronic acid, and 4-O-methylglucuronic acid (MeGlcA) groups. Birch xylan contains MeGlcA groups [3], while oat xylan contains arabinose residues as arabinose xylan [4].

Given the complex structure of xylan, the complete degradation requires the combined action of multiple glycoside hydrolases, including β-D-1,4-endoxylanase β-D-1,4-Xylosidase and β-xylosidase, as well as side chain hydrolases such as α-L-arabinofuranosidase, α-glucuronidase, and acetylxylanesterase [5]. Among these, β-D-1,4-endoxylanase is crucial, acting on the xylan backbone to reduce its polymerization degree and hydrolyze it into oligosaccharides. Typically, β-D-1,4-endoxylanase is referred to as xylanase for short [6].

According to the CAZy classification system (https://www.cazy.org/Glycoside-Hydrolases.html, accessed on 20 June 2025), xylanases are primarily distributed in families GH5, GH8, GH10, GH11, and GH30. Most xylanases belong to the GH10 and GH11 families [7]. Xylanases in the GH11 family consist of a single catalytic domain, exhibit a relatively low molecular mass (generally below 30 kDa), and adopt a tertiary structure characterized by two large β-sheets and one α-helix [8]. Xylanases of GH10 demonstrate superior stability, acid tolerance, and alkaline resistance, and their catalytic domain features a classic (β/α)_8_-barrel structure (also known as a TIM barrel) [9]. GH5 family xylanases are specific to arabinoxylan substrates [6]. The GH8 family mainly consists of low-temperature enzymes, with only six xylanases from this family identified so far [10]. However, there are few reports about GH30 family xylanases, which require the MeGlcA side chain of xylan for catalytic activity [11].

Wild-type xylanases typically exhibit low catalytic efficiency, high production costs, and demanding reaction conditions, which significantly impede their industrial production and application. Directed evolution [12], often referred to as “irrational design”, has emerged as a powerful tool to tackle these challenges. It mimics natural evolutionary processes via artificial selection and in vitro mutagenesis, enabling targeted optimization of enzyme properties without relying on prior structural or functional insights. In contrast to rational design, which requires prior knowledge of protein structure and function, directed evolution generates genetic diversity randomly and selects mutants with desired properties, making it suitable for engineering enzymes with complex or poorly understood structures. Commonly employed techniques include error-prone PCR (EP-PCR), DNA shuffling, in vitro random recombination, enzymatic site-directed mutagenesis, and staggered extension recombination [13]. Among these methods, EP-PCR is the most widely used due to its simplicity and capacity to produce a large number of selectable mutants [14]. For instance, Xiang et al. constructed a xylanase mutant library from Bacillus using EP-PCR and identified mutants with enhanced activity through targeted selection [15]. Wang et al. improved the thermal stability of xylanase from *Thermotoga maritima* and lower its Km value by EP-PCR [16]. Du et al. achieved a two-fold increase in catalytic activity for a xylanase from a gene library through EP-PCR and targeted selection [17].

*D. dadantii* DCE-01, which can simultaneously secrete pectinase, mannanase, and xylanase, is a bacterial strain used for the biorefinery of herbaceous fibers [18]. However, its application in ramie degumming is constrained by the low catalytic activity of xylanase [19]. This study employs error-prone PCR to increase the mutation frequency of xylanase from DCE-01, aiming to develop a mutant with significantly higher enzyme activity to enhance the biological degumming process.

## 2. Materials and Methods

### 2.1. Materials

#### 2.1.1. Bacterial Strains and Plasmids

The vectors and hosts for xylanase gene cloning and protein expression were plasmid pET28a(+) and *Escherichia coli* DH5α and *E. coli* BL21(DE3) (Takara, Dalian, China). pET-28a-*xyn* constructed previously is a vector recombined with the xylanase gene from *D. dadantii* DCE-01 [19].

#### 2.1.2. Chemicals and Media

Taq DNA Polymerase (5 U/μL), MASTERMIX PCR Kit, and T4 Ligase Kit were purchased from TOYOBO (Osaka, Japan). The restriction endonucleases *Bam*HI, *Hind*III, protein marker, dATP, dGTP, dCTP, and dTTP were obtained from Thermo Fisher Scientific (Waltham, MA, USA). DNA marker III was acquired from Tiangen (Beijing, China) of China. OMEGA (San Diego, CA, USA) supplied plasmid mini extraction kit, DNA gel recovery kit, and PCR product purification kit. IPTG (isopropyl thio-β-D-galactoside) and kanamycin were purchased from Promega. Corn xylan, birchwood polysaccharides, oatwood polysaccharides, beechwood polysaccharides, and kanamycin (50 mg/mL) were purchased from Sigma-Aldrich (St. Louis, MI, USA). Pectinase (158 U/mL) was produced by Novozymes (Tianjin, China). The ramie bast fibers were from Institute of Bast Fiber Crops of Chinese Academy of Agricultural Sciences, Changsha, China. China National Pharmaceutical Group Chemical Reagent Company (Beijing, China) provided all other chemical reagents.

LB (Luria Bertani) medium: Tryptone 1%, yeast extract 0.5%, NaCl 1%. Used for the cultivation of *E.coli*.

Screening medium for producing xylanase strains: LB agar medium was supplemented with 0.5% (*w*/*v*) xylan (Sigma). The medium was then adjusted to include kanamycin at a final concentration of 50 μg/mL and IPTG at a final concentration of 1 mM. This medium was employed for the screening of recombinant strains for xylanase production.

Induction medium for recombinant strains: LB medium was supplemented with kanamycin to a final concentration of 50 μg/mL and IPTG to a final concentration of 1 mM. This medium is used for inducing enzyme production in recombinant strains.

### 2.2. Methods

#### 2.2.1. Construction of the Mutant Library

To construct a mutant library, the error-prone PCR method was applied by XYNF (5′-CGggatcc ATGAATGCTATGAATGGAAA-3′) as forward and XYNR (5′-CCaagctt TTATTTACTGCAAAGGTCGT-3′) (lowercase letters are restriction endonucleases *Bam* HI and *Hind* III, respectively) as reverse primers, using plasmid pET-28a-*xyn* as a template. Error-prone random mutagenesis was performed by adding certain concentrations of dNTPs, Mg^2+^, and Mn^2+^ to a standard PCR reaction system. Error-prone PCR mutagenesis system: 10 × PCR buffer 5 μL, add MgCl_2_ to achieve final concentrations of 4 mM, 5 mM, 6 mM, or 7 mM, MnCl_2_ at different final concentrations (0.2 mmol/L/0.3 mmol/L/0.4 mmol/L), dATP 0.2 mmol/L, dGTP 0.2 mmol/L, dCTP 1 mmol/L, dTTP 1 mmol/L, upstream primer 1 μL (10 µM), downstream primer 1 μL (10 µM), template 1 μL, Taq polymerase 0.5 μL, add ddH_2_O to make up 50 μL. After thoroughly mixing the solution of the reaction system, PCR amplification was performed as follows: at 94 °C for 4 min; followed by 30 cycles at 94 °C for 30 s, 56 °C for 30 s, and 72 °C for 2 min.

After the first round of PCR, the PCR products were detected by 1% agarose gel electrophoresis and subsequently recovered using gel recovery kits. The second round of PCR amplification was then performed with the corresponding concentrations of MgCl_2_ and MnCl_2_ from the initial reaction system, following the same PCR amplification system and reaction procedure as the first round.

The PCR product was ligated to vector pET-28a and transformed into *E. coli* DH5α competent cells. Three colonies were randomly selected from each group and sequenced to analyze the mutation frequencies. The best EP-PCR reaction system was determined according to the results of gel electrophoresis of PCR products and mutation rate.

Using the plasmid pET-28a-xyn as template DNA, the xylanase gene (*xyn*) was cloned under the determined optimal EP-PCR conditions. The PCR products were detected by 1% agarose gel electrophoresis and recovered using the gel recovery kit. The recovered EP-PCR fragments were named *xyn-ep*.

The purified *xyn-ep* was digested with *Bam* HI and *Hind* III. The pET-28a plasmid was similarly digested. The double-digested vector and *xyn*-*ep* were ligated using the T_4_ DNA ligase, and then, we constructed the recombinant plasmids for pET-28a-*xyn*-*ep*, containing mutated xylanase gene. The recombinant plasmids for pET-28a-*xyn*-*ep* were transformed into competent *E. coli* BL21 (DE3) cells to construct a mutant library. After culturing, two hundred colonies were randomly selected for PCR amplification, and ten typical single colonies were selected to confirm the successful construction of the mutant library.

#### 2.2.2. Screening of Mutant Strains

The mutants were plated on a screening medium and cultured at 37 °C for 16–24 h. Gram iodine solution was added [20], and the strains were screened based on the diameter of the transparent zone surrounding the colonies. Colonies with larger hydrolysis zones were selected and inoculated into liquid LB medium containing 50 μg/mL kanamycin and 1 mM IPTG. The cultures were incubated at 37 °C for 16–24 h, and enzyme activity was measured. The mutant xylanase with the highest activity was selected for further research.

#### 2.2.3. SDS-PAGE Analysis

Fermentation broth of the recombinant expression strain was centrifuged at 13,000 r/min for 1 min. The supernatant was discarded, and the pellet was washed twice with 500 μL of ddH_2_O. The bacterial cells were re-suspended in 50 μL of ddH_2_O, followed by the addition of an equal volume of 2 × loading buffer. The mixture was boiled in a boiling water bath for 5 min. 10 μL aliquot of the sample was then loaded for sodium dodecyl sulfate polyacrylamide gel electrophoresis (SDS-PAGE) [21]. After electrophoresis, the gel was placed in a culture dish containing fixative and fixed overnight [22]. The fixative was then discarded, and the gel was rinsed with ddH_2_O. Staining solution was added, and the gel was stained for 3–4 h. After staining, the gel was washed 2–3 times with ddH_2_O, decolorizing solution was added for decolorization, and the process continued until the protein bands were clear.

#### 2.2.4. Determination of Xylanase Activity of Mutant Strains

To determine the activity of xylanase, 200 μL of the enzyme solution was mixed with 800 μL of 1% xylan substrate prepared in citric acid-sodium citrate buffer. The mixture was incubated in a 50 °C water bath for 5 min. The released reducing sugars were determined using the DNS method [23]. One unit (U) of xylanase activity was defined as the amount of enzyme required to release xylose at a rate of 1 μmol/min under the given conditions [24].

Enzyme activity (U) = 1000 M × N/(T × L × 150.13)(1)

In the formula, M is the mass of reducing sugar (xylose) (mg), N represents the dilution factor of the enzyme solution, T is the reaction time (min), L is the volume of the reaction enzyme solution (mL), and 150.13 is the relative molecular weight of xylose.

#### 2.2.5. Temperature and Optimal pH of Mutant Xyn-ep

The mutant strain was inoculated into LB induction medium containing 50 μg/mL kanamycin and 1 mM IPTG, followed by shaking cultivation at 37 °C and 180 rpm for 16 h (incubation time). After centrifugation at 8000 rpm for 10 min, the supernatant was collected as the crude enzyme solution. According to Section 2.2.4, the enzyme activity at different temperatures and pH were determined.

The mutant xylanase (Xyn-ep) activity of the enzyme solution was measured at six temperature values: 30 °C, 40 °C, 45 °C, 50 °C, 55 °C, and 60 °C. The highest enzyme activity was set as 100%, and the relative enzyme activity of each sample was calculated accordingly.

The crude enzyme solution was placed in pre-prepared buffer solutions of different pH values: acetic acid-sodium acetate buffer (pH 3.0–4.5), citric acid-sodium citrate buffer (pH 5.0), and phosphate buffer (pH 5.5–8.0). The enzyme solution was incubated at 50 °C for 5 min in each buffer to determine enzyme activity. The highest activity was recorded as 100%, and the relative activity of each sample was calculated.

#### 2.2.6. Thermal Stability of Mutated Xyn-ep

The thermal stability of the mutated enzymes was assessed by incubating the crude enzyme solution at temperatures of 20 °C, 30 °C, 40 °C, 50 °C, 60 °C, and 70 °C for 60 min. The remaining enzyme activity was measured. The enzyme activity after 60 min of incubation at 4 °C was defined as 100%, and the enzyme activity under other conditions was expressed as relative activity.

#### 2.2.7. Substrate Specificity of Mutant Xyn-ep

The degradation ability of the mutant Xyn was tested on various substrates, including 1% (*w*/*v*) solutions of beech xylan, birch xylan, oat xylan, corn xylan, and ramie hemicellulose. Xyn activity was determined by incubating each substrate with the purified enzyme under standard assay conditions.

#### 2.2.8. Structural Analysis of Xyn-ep

The strain with the highest enzyme activity was inoculated into LB liquid medium and cultured for 12–16 h. The plasmid was extracted, digested with two restriction enzymes, and analyzed by gel electrophoresis. The successfully detected plasmid was sent to a sequencing company for sequencing. The sequencing results were compared with the original xylanase gene to analyze mutant sites in nucleotide bases and amino acid residues.

The spatial structure of the mutated xylanase was simulated using the SWISS-MODEL modeling system (https://swissmodel.expasy.org/, accessed on 12 February 2025). Amino acid mutation sites were then analyzed using Pymol software (3.1.0a0). This approach allowed for a detailed understanding of how specific mutations affected the enzyme’s structure and function.

#### 2.2.9. The Degumming Performance of Mutant Strains on Ramie

Although pectin occurs at lower levels in ramie bast than hemicellulosic components such as xylan, it forms a coating around hemicellulose, preventing xylanase from accessing its substrate. To effectively observe the action of xylanase, a pectinase pretreatment was conducted: raw ramie was immersed for 16 h in a 16 g/L Novozymes alkaline pectinase solution at pH 10 and 50 °C.

After pectinase pretreatment, the ramie bast was thoroughly rinsed and added to LB medium at a concentration of 5% (*w*/*v*) and followed by sterilization. Recombinant strains expressing either the wild-type or mutant xylanase were inoculated into the medium at 2% (*v*/*v*). The cultures were incubated at 37 °C with shaking at 180 rpm for 3 h, after which IPTG was added. Fermentation was then continued at 30 °C with shaking at 180 rpm for a further 12 h.

Degummed ramie fibers were processed following the method: the degummed ramie was filtered through a 300-mesh sieve, mechanically beaten and rinsed to remove residual gum, then dried to constant weight at 50 °C. the dried ramie was photographed with a camera to compare the surface morphology; The microstructural alterations at the fiber surface was observed using a scanning electron microscope (SEM, Hitachi SU3500) (Hitachi High-Technologies Corporation, Tokyo, Japan).

The content of reducing sugars was determined by the DNS method. The light absorption value of reducing sugar was OD_540_. Xylose was used as the standard curve to convert the reducing sugar content.

Determination of weight loss rate [25]: The mass of the raw ramie and that of fermented ramie were set as *G_m_* and *G_f_*, respectively, and the weight loss rate was calculated through the following formula:

(2) $$\mathrm{Weight}\;\mathrm{loss}\;\mathrm{rate}\left(\%\right)=\frac{G_{m}-G_{f}}{G_{m}}\times 100$$

## 3. Results and Discussion

### 3.1. Error-Prone PCR Mutagenesis

Standard approaches for error-prone PCR involve modifying the dNTP concentrations in the PCR system, increasing Mg^2+^ levels, and incorporating specific concentrations of Mn^2+^. Elevated Mg^2+^ concentrations help stabilize non-complementary base pairs, while Mn^2+^ primarily reduces the polymerase’s specificity to the template. In our study, the final concentrations of dATP and dGTP were set at 0.2 mmol/L (mM), while dCTP and dTTP were maintained at 1 mM. We explored suitable error-prone PCR conditions for Mn^2+^ concentration based on typical Mg^2+^ concentrations. Mg^2+^ concentrations were tested at 4, 5, 6, and 7 mM, while Mn^2+^ concentrations were set at 0.2, 0.3, and 0.4 mM. The combinations of these ion concentrations are shown in Table 1.

**Figure 1:**
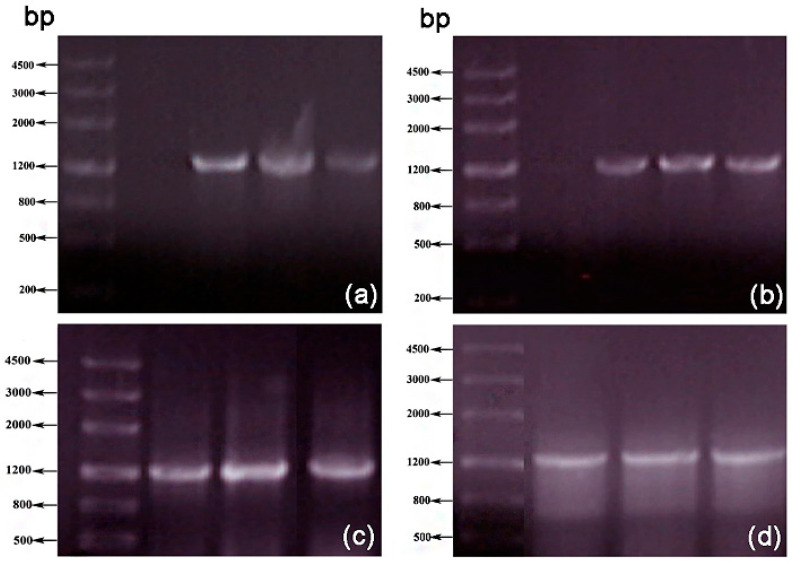
Electrophoresis of error-prone PCR at different Mg^2+^ ionic concentrations: (**a**) 4 mmol/L, (**b**) 5 mmol/L, (**c**) 6 mmol/L, and (**d**) 7 mmol/L. Note: On each gel, the manganese ion concentrations in the three lanes from left to right are 0.2 mM, 0.3 mM, and 0.4 mM, respectively.

Electrophoresis of the PCR products (Figure 1) showed that a DNA fragment of approximately 1.2 kb could be amplified across various Mg^2+^ and Mn^2+^ concentrations, matching the target fragment size. However, Groups 10, 11, and 12, with 7 mM Mg^2+^ (Figure 1d), exhibited dispersed bands. Groups 1–9 produced clear results, under the concentration of 5 mM MgCl_2_, the bands of the three groups (Figure 1b) are clear. PCR products from Groups 1–9 were ligated into vectors and transformed into DH5α competent cells. After cultivation, three colonies from each group were randomly selected for sequencing to determine the mutation rate.

**Figure 2:**
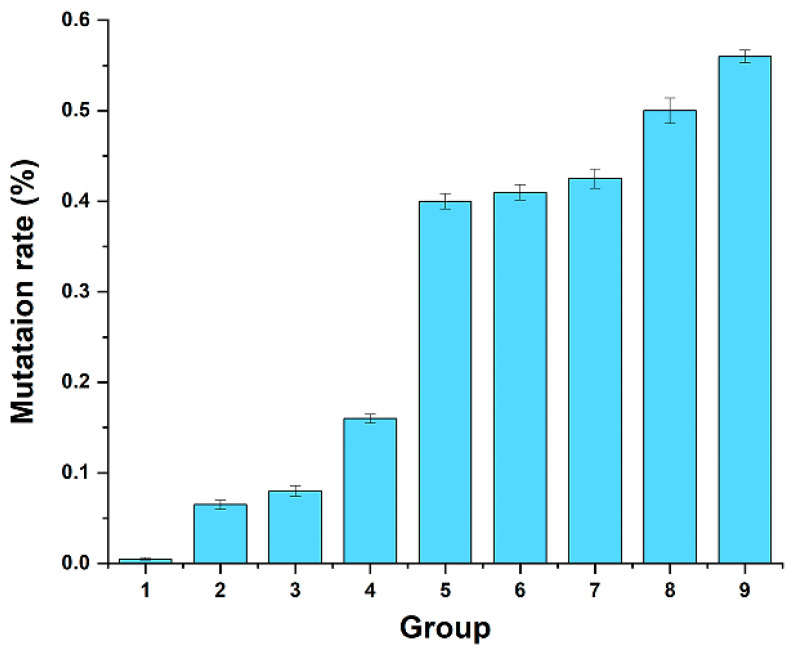
Mutation rate of error-prone PCR of different groups. The concentrations of Mg^2+^ and Mn^2+^ in Groups 1–9 are as follows: Group 1: Mg^2+^ 4 mM, Mn^2+^ 0.2 mM; Group 2: Mg^2+^ 4 mM, Mn^2+^ 0.3 mM; Group 3: Mg^2+^ 4 mM, Mn^2+^ 0.4 mM; Group 4: Mg^2+^ 5 mM, Mn^2+^ 0.2 mM; Group 5: Mg^2+^ 5 mM, Mn^2+^ 0.3 mM; Group 6: Mg^2+^ 5 mM, Mn^2+^ 0.4 mM; Group 7: Mg^2+^ 6 mM, Mn^2+^ 0.2 mM; Group 8: Mg^2+^ 46 mM, Mn^2+^ 0.3 mM; and Group 9: Mg^2+^ 6 mM, Mn^2+^ 0.4 Mm.

Figure 2 presents the base mutation rate for each group, emphasizing the relationship between different concentrations of Mg^2+^ and Mn^2+^ and the base mutation rate. No mutations were detected in any of the three recombinant colonies within Group 1. Group 2 had one colony with a single base change, while Group 3 had one colony with two base changes. The number of mutated bases in Group 4 ranged from 1 to 3, while Group 5 exhibited mutation counts of 3, 6, and 6 across three colonies. Groups 6 through 9 averaged between 5 and 7 mutations per colony. Based on these mutation rates, Groups 5, 6, and 7 were identified as suitable for constructing a mutation library. Consequently, the reaction system of Group 5 (Mg^2+^: 5 mM, Mn^2+^: 0.3 mM) was chosen for further experiments.

**Figure 3:**
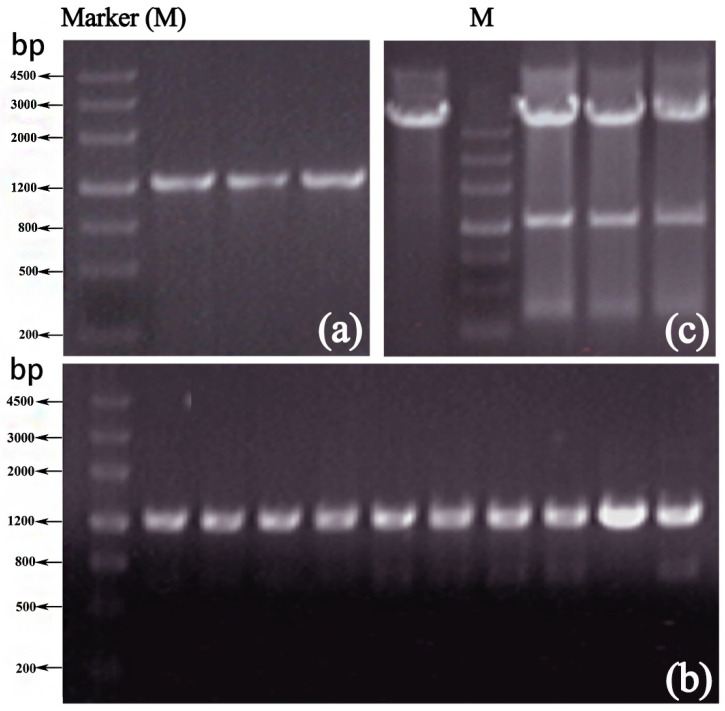
PCR amplification of *xyn*-*ep* and restriction enzyme digestion of the recombinant plasmid. (**a**) Error-prone PCR product; (**b**) PCR detection of recombinant plasmids; (**c**) double-enzyme digestion of recombinant plasmids.

Using the recombinant plasmid pET-28(a)-xyn as a template, two rounds of PCR amplification of the xylanase gene were performed under conditions of 5 mmol/L Mg^2+^ and 0.3 mmol/L Mn^2+^ using an error-prone PCR system. Results (Figure 3a) revealed clear bands with a cloned fragment size of approximately 1.2 kb, consistent with the size of the xylanase gene. The mutant fragment of the xylanase gene was designated as *xyn-ep*.

The error-prone PCR products were purified via gel recovery, digested, ligated, and transformed into competent *E. coli* BL21 (DE3) to construct a mutant library of xylanase genes. After 16 h of cultivation at 37 °C, 10 randomly selected transformed single colonies were subjected to PCR amplification. As depicted in Figure 3b, all 10 colonies amplified products of the same size as the xyn fragment, confirming that all recombinant plasmids contained mutated xylanase gene fragments and that the mutant Xyn library was successfully constructed.

**Figure 4:**
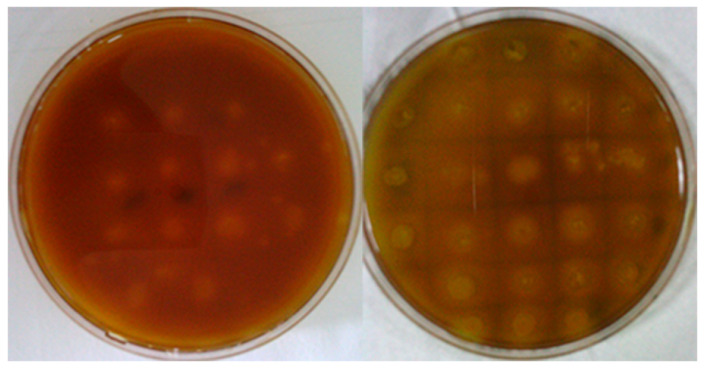
Screening plate of mutant strains. The xylanase activity of these 40 strains was measured at 50 °C after inducing enzyme production in liquid culture medium. Seven strains with higher enzyme activity than the original xylanase were identified (Figure 5). Among them, the Xyn-ep mutant strain exhibited 1.6 times higher enzyme activity than the control strain.

Transformants were plated on a screening medium to establish an expression library of mutant genes. After 24 h of cultivation, iodine solution was added to screen for 40 mutant strains with larger transparent zones (Figure 4).

High-throughput screening of mutant xylanase is often performed using 96-well plates [26]. The 96-well plate high-throughput screening method offers the advantages of processing multiple samples simultaneously and saving a significant amount of reagents. However, the 96-well plate screening method also has its limitations: after cultivation of the mutants, subsequent centrifugation and enzyme activity measurements are still required, which increases the complexity of the experiment, and it is difficult to intuitively judge the level of enzyme activity after mutant cultivation. The spread plate method [14,27] has been used for screening to overcome the limitations of 96-well plates screening. This approach allows for a more intuitive assessment of enzyme activity through the formation of hydrolysis zones, simplifying the selection process for high-activity mutants. In this study, the screening of mutants was carried out using the plate spreading and iodine staining methods, which provides intuitive results. After cultivation, mutants can be directly selected for further studies based on the size of the hydrolysis zones. This approach simplifies the screening process and allows for rapid identification of high-activity mutants.

**Figure 5:**
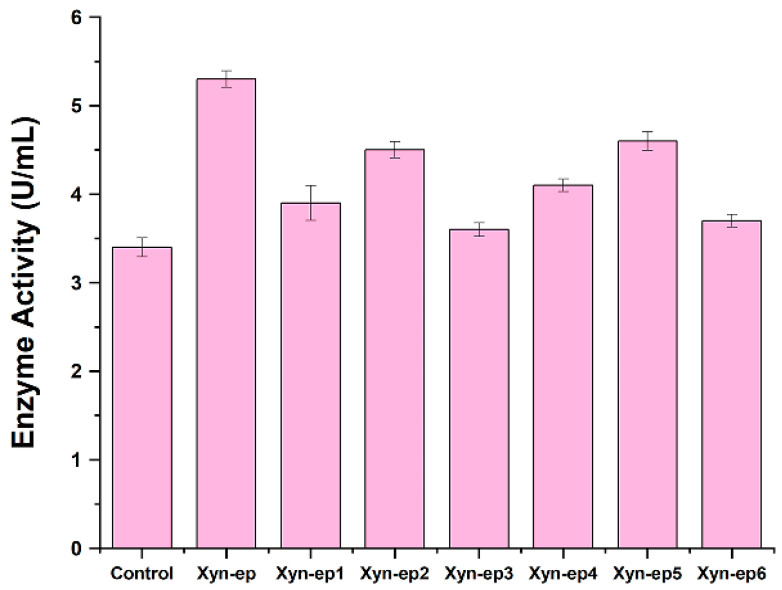
Xylanase activity of control strain and mutual strains.

**Figure 6:**
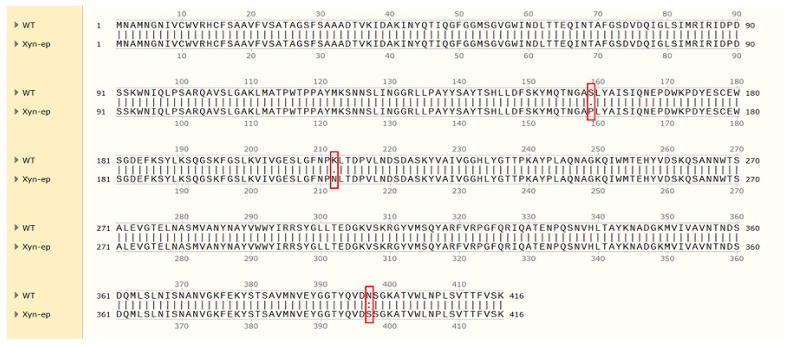
Amino acid sequence alignment of WT and Xyn-ep (Red boxes highlight the three key amino acid mutation sites: S159P, K212N, N397S).

The Xyn-ep recombinant strain was cultured in LB medium, and plasmids were extracted and verified by double-enzyme digestion. Compared with the empty vector (Figure 3c), the recombinant plasmid was found to contain a mutated xylanase gene, confirming its successful recombination into the expression vector. The gene size is approximately 1.2 kb, similar to the original gene. The recombinant plasmid was verified and sent for sequencing. The sequencing results indicated four point mutations: 156A to G, 475 T to C, 636A to T, and 1190A to G. Alignment of the translated amino acid sequence from the mutated gene with the parent protein sequence (as shown in Figure 6) revealed three mutations: serine at position 159 to proline (S159P), lysine at position 212 to asparagine (K212N), and asparagine at position 397 to serine (N397S). The mutation at position 156 resulted in a nonsense mutation.

### 3.2. Analysis of Enzymatic Properties of Mutant Strains

**Figure 7:**
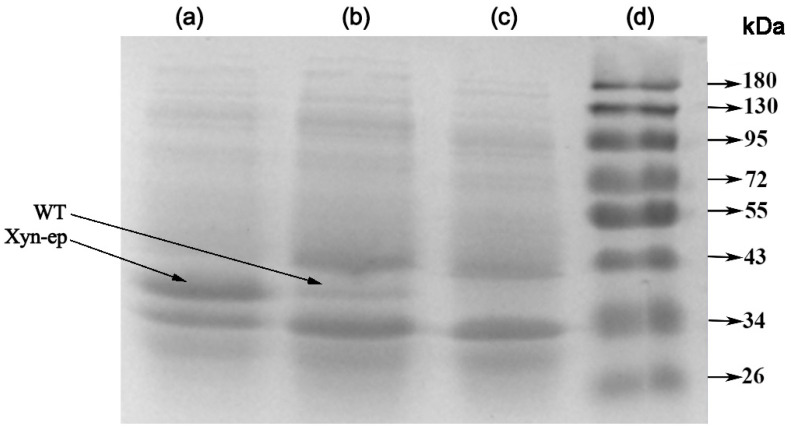
SDS-PAGE results of recombinant strains. (**a**) BL21(DE3)-pET-28a(+)-Xyn-ep; (**b**) BL21(DE3)-pET-28a(+)-Xyn; (**c**) BL21(DE3)-pET-28a(+) and (**d**) protein molecular standard (kDa).

SDS-PAGE analysis of the proteins (Figure 7) revealed an additional protein band in the experimental group between 34–43 kDa compared to the blank control group. The Xyn-ep had the same molecular weight as the parental type, with both protein bands appearing at the same level. Under the same conditions, the band for the mutant xylanase was significantly wider than that of the parental type, indicating that Xyn-ep had a significantly higher expression level than the parent enzyme.

**Figure 8:**
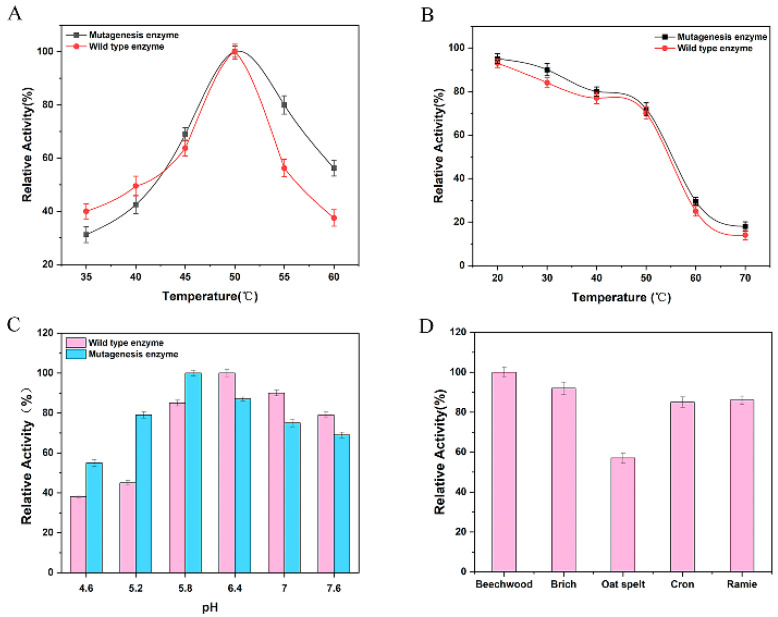
Enzymatic properties of mutant Xyn. (**A**) The optimum temperature; (**B**) thermal stability; (**C**) optimum pH; and (**D**) substrate specificity.

Generally, temperature and pH are two key factors influencing xylanase activity. The trend of relative enzyme activity (The highest enzyme activity was set as 100%, and relative enzyme activities at different temperatures were calculated) with temperature variation is shown in Figure 8A. The results indicated that the optimal temperature for the mutant Xyn-ep was 50 °C, the same as for the wild-type enzyme (parent enzyme). However, the enzyme activity remained above 70% within the temperature range of 45–55 °C, whereas the parental type exhibited a sharp decline in activity above 50 °C. This suggests that the mutant Xyn has a broader temperature adaptation range than the parental enzyme.

To assess thermal stability, the crude enzyme solution of the mutant Xyn was incubated at temperatures ranging from 20 °C to 70 °C for 60 min, and enzyme activity was measured. Enzyme activity after 60 min of incubation at 4 °C was set as 100%, with activities under other conditions expressed as relative values. Results (Figure 8B) showed that the thermal stability of the mutant Xyn was similar to that of the parent enzyme, and both decreased with increasing temperature. When the temperature exceeded 50 °C, the thermal stability sharply declined.

To determine the effect of pH on enzyme activity, 1% xylan substrate from beech wood was prepared with buffer solutions of varying pH values, and the activity of the mutant xylanase Xyn-ep was measured at 50 °C. Results (Figure 8C) showed that the optimal pH for the mutant xylanase was approximately 5.8, lower than that of the wild-type enzyme. Within the pH range from 5.2 to 7.6, Xyn-ep maintained at least 70% of its maximum activity, demonstrating a wider pH tolerance and higher acid–base stability than the parental type.

The substrate specificity of Xyn-ep was also evaluated. Results (Figure 8D) indicated that beech wood xylan and birch wood xylan were the most suitable substrates for the Xyn-ep. The mutant xylanase also exhibited high activity on ramie hemicellulose, reaching over 80% of the activity observed with the optimal substrate. These findings suggest that the Xyn-ep has a broad substrate specificity, making it suitable for processing various types of xylan substrates.

### 3.3. Structural Analysis of Mutated Enzymes

**Figure 9:**
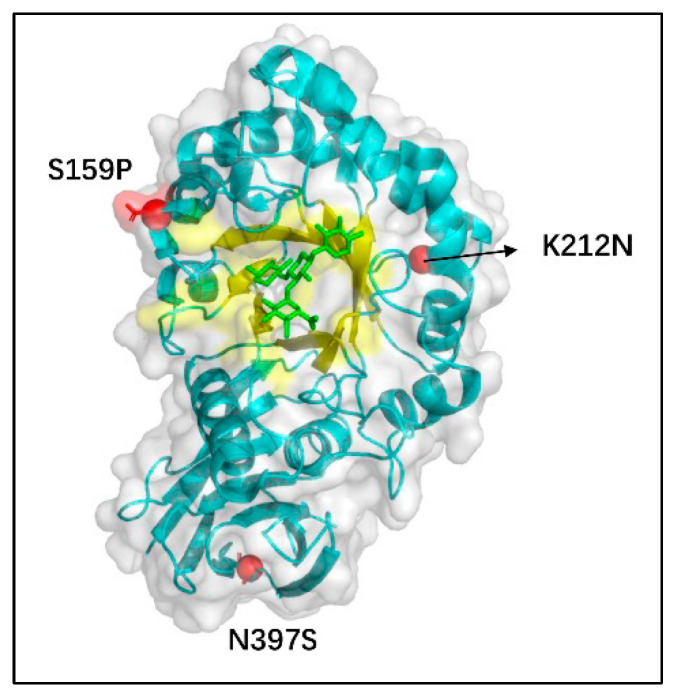
Three-dimensional structural model of mutant xylanase. Three red spheres represent the mutation sites, the yellow area indicates the protein’s active center, and the blue part is the protein backbone.

To elucidate the spatial configuration of the Xyn-ep, the Swiss Model server was employed to simulate its three-dimensional structure. This simulation was based on the protein crystal structure 1NOF (PDB: 2A2Y) from the Protein Data Bank, achieving a similarity of 92%, which underscores the reliability of the simulation results. Structural analysis (Figure 9) shows that the mutation site S159P is situated on the loop of the catalytic domain, K212N is located on the protein surface within the alpha helix of the catalytic domain, and N397S is on the loop of the XBM structure.

**Figure 10:**
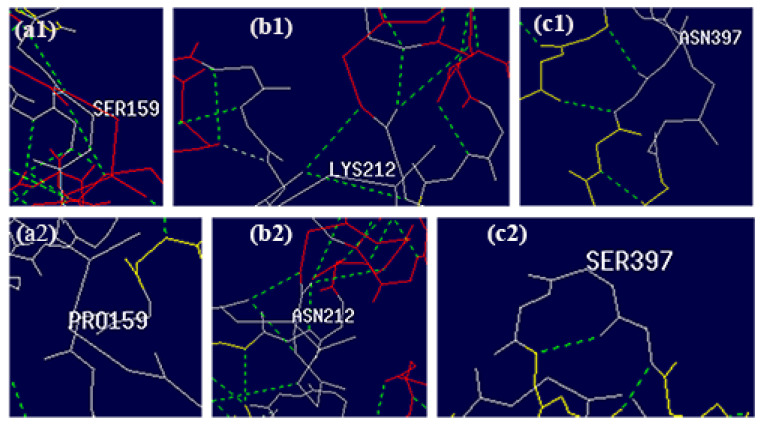
Hydrogen bonds around the amino acid residues of 159th, 212th, and 397th positions (Green dashed lines consistently represent hydrogen bonds across all subpanels): (**a1**) WT structure at residue Ser159, (**a2**) Mutant structure at residue Pro159 (S159P), (**b1**) WT structure at residue Lys212, (**b2**) Mutant structure at residue Asn212 (K212N), (**c1**) WT structure at residue Asn397, (**c2**) Mutant structure at residue Ser397 (N397S).

The S159P mutation likely arises from the absence of a hydroxyl hydrogen on the side chain, which prevents hydrogen bond formation with other amino acids (Figure 10(a2)). This increases the flexibility of the loop, making the structure more dynamic. In contrast, serine is a nucleophilic residue that readily forms hydrogen bonds (Figure 10(a1)). The conversion of serine to proline may reduce steric hindrance because that proline, with its pyrrolidine ring structure, has limited conformational freedom [28], facilitating the rotation of adjacent alpha helices and bringing them closer to the active center.

K212N is located on an alpha helix on the protein surface. As depicted in Figure 10(b1,b2), the mutation from lysine to asparagine increases the number of surrounding hydrogen bonds, potentially stabilizing the catalytic domain and thereby enhancing enzyme activity.

N397S is situated on the loop of the binding domain. Figure 10(c1,c2) indicate that the mutation from aspartic acid to serine reduces hydrogen bonding at position 397, increasing loop flexibility and facilitating closer interaction between the binding and catalytic domains, thus improving catalytic efficiency.

Among various mutation methods, EP-PCR stands out as a simple, cost-effective and convenient approach [29] that generates diverse mutations without requiring prior knowledge of protein structure. This overcomes the limitations of rational design [30]. Recent studies have successfully used EP-PCR to modify xylanase properties [31]. However, due to the non-directional nature and multiple types of mutations, this method requires screening thousands of mutants, extending the cycle. Additionally, polymerase mutations exhibit preferences, leading to low frequencies of insertions or deletions in error-prone PCR reactions [32]. Most mutations are point mutations, often involving base transitions with low mutation rates [33], which also reduces the diversity. In this study, all observed mutations were point mutations, predominantly adenine substitutions, consistent with most research findings [34].

The success of error-prone PCR hinges on controlling the mutation rate. Generally speaking, a high mutation rate can deactivate xylanase, while a low rate may yield too many parental enzymes. Chirumamilla et al. [35] suggest that an ideal mutation rate for enzyme directed evolution is 2–7 nucleotide mutations per gene. Thus, optimizing the mutation rate is crucial for successful directed evolution of xylanase. Mutation frequency in error-prone PCR is influenced by factors such as ion concentration, gene length, PCR cycle parameters, and polymerase fidelity [36]. Different gene sequences can produce varying mutation frequencies under identical conditions, underscoring the need for tailored error-prone PCR designs. By exploring Mg^2+^ and Mn^2+^ concentrations, it was determined that Group 5 had a 0.4% mutation rate and an average of five mutated bases (Figure 2), which was suitable for enzyme directed evolution. This establishes a robust mutational response system that enables two rounds of error-prone PCR to generate a diverse library of mutants and improve the efficiency of directed evolution. However, the changes in amino acids of the mutated enzyme via error-prone PCR were either on the protein surface (K212N) or in loop regions (S159P and N397S) far from the active center and have little impact on catalytic activity [37]. Consequently, improvements in catalytic activity and other properties of the obtained mutated enzyme were not significant. Voigt et al. similarly observed that random mutations often occur on protein surfaces or loops [38]. The mutation occurring at the loop may increase the flexibility of the loop, improve the elasticity of the enzyme’s spatial structure, facilitate the binding of substrates and catalytic centers, and thus boost catalytic activity. Protein surface mutations may stabilize the catalytic structure, aiding the recognition of substrates by active sites. However, multiple amino acid mutations can have additive effects, causing interactions or changes across different domains of the entire enzyme molecule, potentially altering activity or stability.

### 3.4. Effect of the Mutant Xylanase on Ramie Degumming

**Figure 11:**
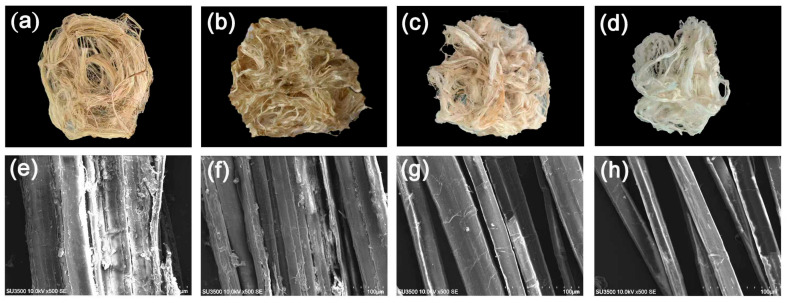
Surface morphology of the bio-degummed ramie. Photographs of (**a**) negative control, (**b**) pectinase pretreatment, (**c**) pectinase + Xyn and (**d**) pectinase + Xyn-ep; SEM images of (**e**) negative control, (**f**) pectinase pretreatment, (**g**) pectinase + Xyn and (**h**) pectinase + Xyn-ep.

The macroscopic appearance of ramie fibers can intuitively reflect the efficiency of degumming, as shown in Figure 11a–d. Among these, Figure 11a represents the negative control, Figure 11b the pectinase pretreatment group, while Figure 11c,d display ramie fibers fermented by strains Xyn and Xyn-ep, respectively. Notably, the fibers treated with Xyn-ep are significantly more dispersed, supple, and glossy compared to both the control and Xyn-treated groups.

Scanning electron microscopy was used to observe the surface morphology of degummed ramie, with results presented in Figure 11e–h. As shown in Figure 11e, the surface of raw, non-degummed ramie is rough, with a substantial amount of gum covering the fiber surface and causing fibers to adhere together. After pectinase pretreatment (Figure 11f), gum fragments are reduced, and the fibers are dispersed. Following treatment with wild-type xylanase (Figure 11g), most of the gum is removed, the fiber surfaces are relatively smooth, and the fibers show good dispersion. After fermentation of ramie with recombinant strains containing the mutant enzyme (Figure 11h), the gum is completely removed, the fiber surfaces are smooth, and the fibers are finer. These results indicate the degumming effect of the mutant enzyme is higher than that of the wild-type enzyme.

The ramie weight loss rates and reduction in sugar content after different enzyme treatments were determined. Results in Table 2 showed that the weight loss rate of ramie treated by mutant Xyn-ep was highest (19.73%), and that of wild type Xyn treatment was 15.72%. The weight loss rate of ramie treated by mutant Xyn-ep was 0.149 mg/mL, higher than that of the wild type Xyn treatment (0.116 mg/mL).

## 4. Conclusions

Error-prone PCR was employed to modify GH30 xylanase from *D. dadantii* DCE-01, resulting in the determination of an optimal PCR reaction system and the establishment of a method for screening mutants using iodine solution to visualize hydrolysis circles. Seven gene-engineering strains with high activity were obtained, and a mutant exhibited significantly higher activity than *D. dadantii* DCE-01 following rescreening. The expressed xylanase retained substrate specificity and optimal reaction temperature similar to that of *D. dadantii* DCE-01, while its optimal pH value decreased and acid–base tolerance improved. The mutated enzyme featured three amino acid substitutions: serine at 159th position to proline (S159P), lysine at 212th position to asparagine (K212N), and asparagine at 397th position to serine (N397S). Structural simulation revealed that the K212N mutation is located on the protein surface within the catalytic domain far from the catalytic center, while the other two mutations occur in loop regions. This indicated that error-prone PCR is an effective method for enhancing enzyme activity, and the mutant Xyn-ep may be a new GH30 xylanase, being a potential candidate for industrial applications such as bast fiber bio-degumming, cotton bio-refinery, paper making, and so on.

## Figures and Tables

**Table 1:** Groups of error-prone PCR (mM).

	Mg^2+^	4	5	6	7
Mn^2+^					
0.2		1	4	7	10
0.3		2	5	8	11
0.4		3	6	9	12

**Table 2:** Comparison of weight loss rates and reducing sugar content of ramie.

	Negative Control	Pectinase Pretreatment	Pectinase + Wild Type Xyn	Pectinase + Mutant Xyn-ep
Reducing sugar content (mg/mL)	0.003	0.512	0.116	0.149
Weight loss rates (%)	4.77	10.89	15.72	19.73

## Data Availability

Data is contained within the article: The original contributions presented in this study are included in the article. Further inquiries can be directed to the corresponding authors.
